# Supplementary material for: Hippocampal-neocortical interactions sharpen over time for predictive actions
Source: Nat Commun. 2019 Sep 5;10:3989. doi: 10.1038/s41467-019-12016-9 (PMC6728336; doi:10.1038/s41467-019-12016-9)
Supplement: Supplementary file 1 — Supplementary Information [file 41467_2019_12016_MOESM1_ESM.pdf]

Supplementary Information

Hippocampal-neocortical interactions sharpen over time for predictive actions

Hindy, Avery, & Turk-Browne

Supplementary Figures 1-3

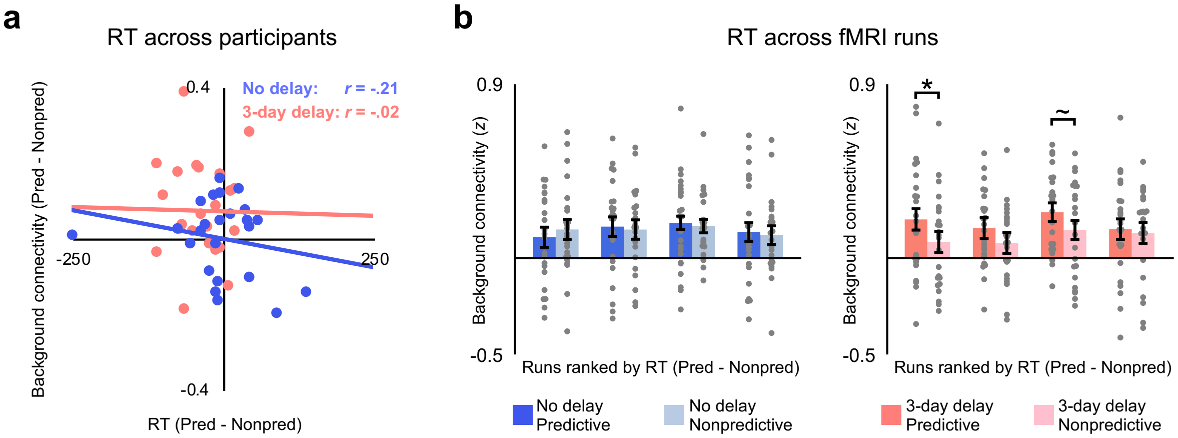

Supplementary Figure 1. RT vs. background connectivity. (a) Individual differences across participants in RT (Predictive – Nonpredictive) were unrelated to differences in background connectivity immediately after training ($r(22) = -.21$, $p = .33$) and after the 3-day delay ($r(22) = -.02$, $p = .94$). **(b)** In order to examine background connectivity across fMRI runs as a function of the RT, we ranked the runs for each participant based on the RT difference between predictive and nonpredictive actions before plotting background connectivity. For each delay condition, fMRI runs are ranked such that the RT was usually slower for predictive vs. nonpredictive actions for the leftmost columns, and usually faster for predictive than nonpredictive actions for the rightmost columns. In repeated measures ANOVAs based on the ranked fMRI runs, predictiveness did not interact with the RT-rank either immediately after training ($F(1, 23) = 0.38$, $p = .55$) or after the 3-day delay ($F(1, 23) = 1.29$, $p = .27$). Error bars indicate ±1 SEM of the difference between predictive and nonpredictive actions for each run. $*p < .05$; $\sim p < .1$ (paired t-tests). Source data are provided as a Source Data file.

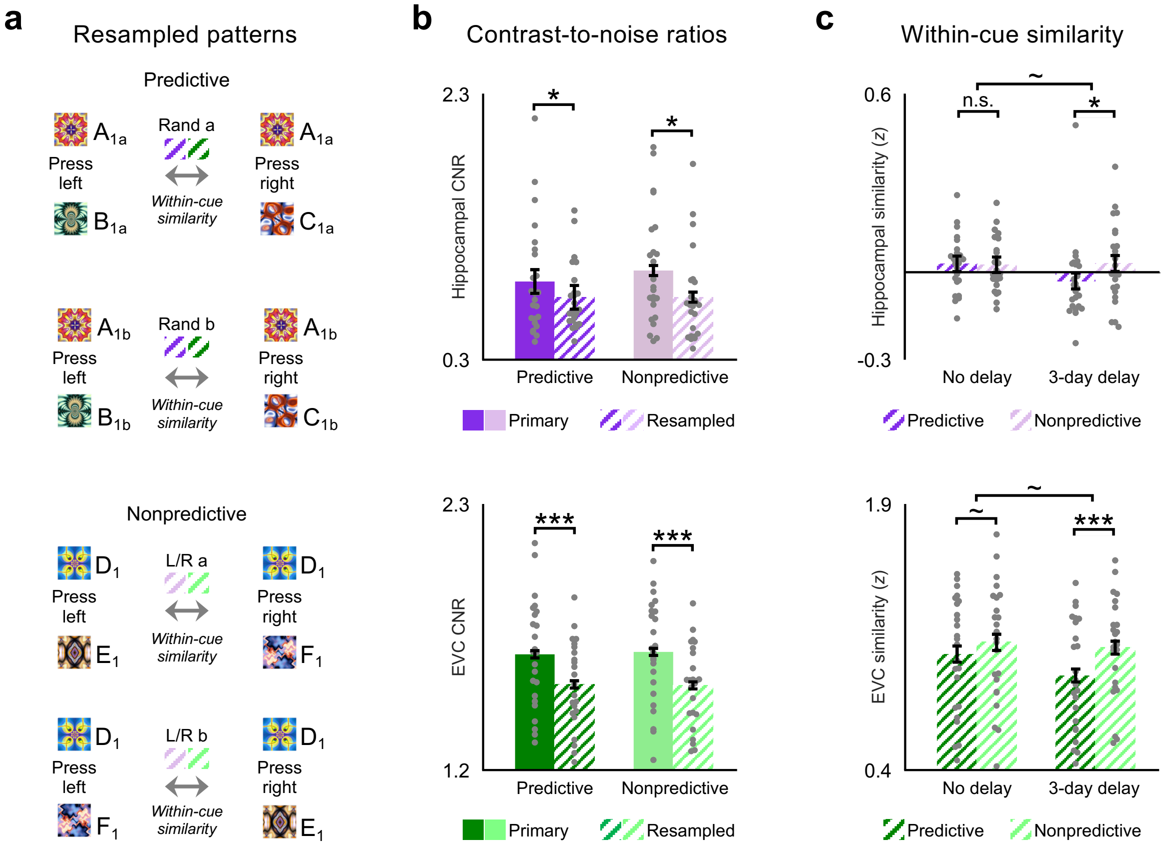

Supplementary Figure 2. Resampled pattern similarity and contrast-to-noise ratios. (a) In primary analyses of pattern similarity among cue-outcome visual transitions (Fig. 7), we averaged across left and right button presses for visual transitions with nonpredictive actions but not predictive actions. To ensure that averaging in this way did not bias the primary findings, here we calculated the within-cue similarity between visual transitions with left vs. right nonpredictive button presses. Additionally, in order to calculate within-cue similarity in the same way for visual transitions with predictive actions, we randomly resampled trials with predictive actions as belonging to either of two partitions ('a' or 'b'). **(b)** We calculated the contrast-to-noise ratio across voxels (CNR = $\sqrt{(\bar{x}^2/\sigma^2)}$) of patterns entered into each analysis. Compared to the primary analyses, CNR of the resampled patterns was significantly lower in both the hippocampus ($F(1, 23) = 15.32$, $p < .001$) and EVC ($F(1, 23) = 57.75$, $p < .001$). Within each analysis, however, CNR did not differ between predictive and nonpredictive events (*p*s > .36). **(c)** Within-cue pattern similarity was lower overall among resampled patterns than in the primary analyses. However, differences in pattern similarity among the conditions were similar to those observed in the primary analysis of within-cue pattern similarity, including marginally reliable interactions between timescale and predictiveness in both the hippocampus ($F(1, 23) = 3.39$, $p = .08$) and EVC ($F(1, 23) = 2.97$, $p = .10$). In the hippocampus, within-cue similarity did not differ among visual transitions trained immediately before the scan ($t(23) = 0.15$, $p = .88$), but was reliably lower for predictive events after a 3-day delay ($t(23) = 2.56$, $p = .18$). In EVC, within-cue similarity was marginally lower for predictive vs. nonpredictive events immediately after training ($t(23) = 1.78$, $p = .09$), and reliably lower after the 3-day delay ($t(23) = 4.53$, $p < .001$). Error bars indicate ±1 SEM of the difference between predictive and nonpredictive actions at each timescale. ***$p < .001$; *$p < .05$; ~$p < .1$ (paired *t*-tests). Source data are provided as a Source Data file.

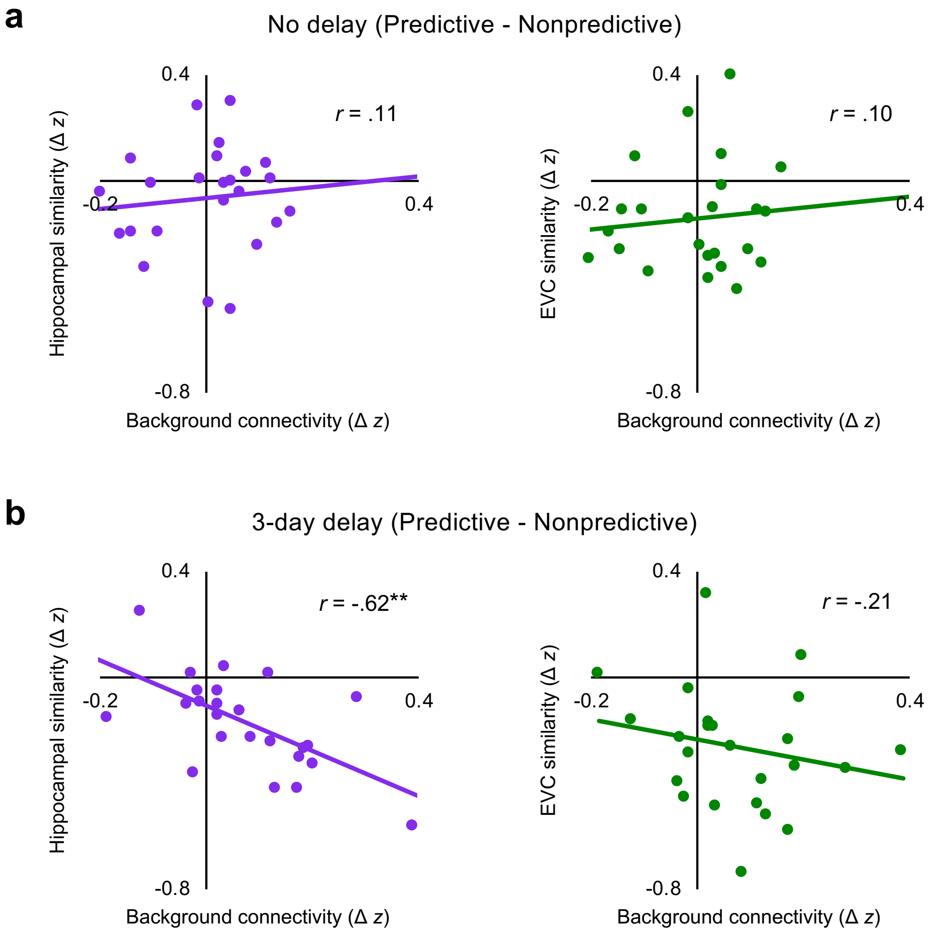

Supplementary Figure 3. Background connectivity vs. within-cue similarity. (a) Immediately after training, differences in background connectivity between predictive and nonpredictive actions were not reliably correlated across participants with the within-cue pattern similarity of either the hippocampus ($r(22) = .11$, $p = .62$) or EVC ($r(22) = .10$, $p = .63$). **(b)** After the 3-day delay, the differences in background connectivity between predictive and nonpredictive actions were negatively correlated across participants with the within-cue pattern similarity of the hippocampus ($r(22) = -.62$, $p = .001$) but not EVC ($r(22) = -.21$, $p = .32$). $^{**}p < .01$ (Pearson correlation coefficient). Source data are provided as a Source Data file.
