# Supplementary material for: Hippocampal-neocortical interactions sharpen over time for predictive actions
Source: Nat Commun. 2019 Sep 5;10:3989. doi: 10.1038/s41467-019-12016-9 (PMC6728336; doi:10.1038/s41467-019-12016-9)
Supplement: Supplementary file 3 — Reporting Summary [file 41467_2019_12016_MOESM3_ESM.pdf]

nature research

Corresponding author(s): Nicholas C Hindy

Last updated by author(s): Aug 11, 2019

Reporting Summary

Nature Research wishes to improve the reproducibility of the work that we publish. This form provides structure for consistency and transparency in reporting. For further information on Nature Research policies, see Authors & Referees and the Editorial Policy Checklist .

Statistics

For all statistical analyses, confirm that the following items are present in the figure legend, table legend, main text, or Methods section.

n/a	Confirmed	
☐	☒	The exact sample size (n) for each experimental group/condition, given as a discrete number and unit of measurement
☐	☒	A statement on whether measurements were taken from distinct samples or whether the same sample was measured repeatedly
☐	☒	The statistical test(s) used AND whether they are one- or two-sided<br>*Only common tests should be described solely by name; describe more complex techniques in the Methods section.*
☐	☒	A description of all covariates tested
☐	☒	A description of any assumptions or corrections, such as tests of normality and adjustment for multiple comparisons
☐	☒	A full description of the statistical parameters including central tendency (e.g. means) or other basic estimates (e.g. regression coefficient) AND variation (e.g. standard deviation) or associated estimates of uncertainty (e.g. confidence intervals)
☐	☒	For null hypothesis testing, the test statistic (e.g. F, t, r) with confidence intervals, effect sizes, degrees of freedom and P value noted<br>*Give P values as exact values whenever suitable.*
☒	☐	For Bayesian analysis, information on the choice of priors and Markov chain Monte Carlo settings
☒	☐	For hierarchical and complex designs, identification of the appropriate level for tests and full reporting of outcomes
☐	☒	Estimates of effect sizes (e.g. Cohen's d, Pearson's r), indicating how they were calculated

Our web collection on statistics for biologists contains articles on many of the points above.

Software and code

Policy information about availability of computer code

Data collection	The Psychophysics Toolbox for MATLAB (MathWorks) was used for stimulus presentation and response collection. fMRI data was collected using CMRR multi-band accelerated EPI pulse sequences.
Data analysis	fMRI data analysis was conducted using FSL 5.0 (FMRIB) and MATLAB (MathWorks)

For manuscripts utilizing custom algorithms or software that are central to the research but not yet described in published literature, software must be made available to editors/reviewers. We strongly encourage code deposition in a community repository (e.g. GitHub). See the Nature Research guidelines for submitting code & software for further information.

Data

Policy information about availability of data

All manuscripts must include a data availability statement. This statement should provide the following information, where applicable:
- Accession codes, unique identifiers, or web links for publicly available datasets
- A list of figures that have associated raw data
- A description of any restrictions on data availability

The source data underlying Figs 1d, 2a–c, 3b–c, 5b–c, 6a–b, and 7a–b and Supplementary Figs 1a–b, 2b–c, and 3a–b is provided as a Source Data file. All neuroimaging data and experimental stimuli are freely available through the OpenNeuro platform for sharing neuroscience data (OpenNeuro.org) with DOI 10.18112/openneuro.ds001946.v1.0.0 [https://openneuro.org/datasets/ds001946/versions/1.0.0].

Field-specific reporting

Please select the one below that is the best fit for your research. If you are not sure, read the appropriate sections before making your selection.

Life sciences study design

All studies must disclose on these points even when the disclosure is negative.

Sample size	The sample size (24 participants) was chosen to match previous fMRI studies with similar behavioral protocols.
Data exclusions	Two additional participants completed the training sessions but did not participate in the fMRI component of the experiment due to below-criterion accuracy on verbal outcome-identification tests prior to the scan.
Replication	All attempts at replication of primary findings within the data set were successful, including ROI analyses split by hemisphere and nonparametric voxelwise analyses permuted within the acquired field of view.
Randomization	For each participant, stimulus images were randomly assigned to be cues or outcomes. We counterbalanced across participants the assignment of images to 3-day delay and no delay conditions and to sequences containing either predictive or nonpredictive actions.
Blinding	Data collection and analysis were not performed blind to the conditions of the experiment. Blinding was not critical since all experimental manipulations were within-subject.

Reporting for specific materials, systems and methods

We require information from authors about some types of materials, experimental systems and methods used in many studies. Here, indicate whether each material, system or method listed is relevant to your study. If you are not sure if a list item applies to your research, read the appropriate section before selecting a response.

Materials & experimental systems

n/a	Involved in the study	
☒	☐	Antibodies
☒	☐	Eukaryotic cell lines
☒	☐	Palaeontology
☒	☐	Animals and other organisms
☐	☒	Human research participants
☒	☐	Clinical data

Methods

n/a	Involved in the study	
☒	☐	ChIP-seq
☒	☐	Flow cytometry
☐	☒	MRI-based neuroimaging

Human research participants

Policy information about studies involving human research participants

Population characteristics	Twenty-four individuals (19 female, aged 18–33 years) from the Princeton University community participated in the study. Each participant was right-handed and had normal or corrected-to-normal vision.
Recruitment	Participants were recruited based on their participation in prior behavioral and fMRI studies at the Princeton Neuroscience Institute. Participants were paid $20 per hour.
Ethics oversight	Princeton University Institutional Review Board

Note that full information on the approval of the study protocol must also be provided in the manuscript.

Magnetic resonance imaging

Experimental design

Design type	Mixed block/event-related design
Design specifications	A total of 320 sequence trials were organized into eight 6-minute runs. Each run contained sequences from either the first training session or the second training session, alternating between runs. Within each run, four blocks of predictive actions alternated with four blocks of nonpredictive actions. Pairs of runs for each participant contained the same stimuli and block order, with the trial order randomized. Each block included five trials and lasted 22.5 s, followed by 18 s of fixation. Within each block, each trial included three parts: a cue stimulus for 1000 ms, an action prompt consisting of a double-headed arrow below the cue that remained on screen until a button press or until the 1500 ms response window elapsed, and an outcome stimulus for 1000 ms.
Behavioral performance measures	Throughout training and in the scanner, we measured choice RT as the time it took for participants to press the left or right button in response to a cue. Additionally, in verbal tests outside the scanner, participants spoke aloud either "top" or "bottom" to indicate which outcome was expected given the cue and action.

Acquisition

Imaging type(s)	Structural and functional
Field strength	3T
Sequence & imaging parameters	Structural and functional MRI data were collected on a 3T Siemens Skyra scanner with a 20-channel head coil. Structural data included a T1-weighted magnetization prepared rapid acquisition gradient-echo (MPRAGE) sequence (1 mm isotropic) for registration and segmentation of EVC, and two T2-weighted turbo spin-echo (TSE) sequences (0.44 × 0.44 × 1.5 mm) for hippocampal segmentation. Functional data consisted of T2*-weighted multi-band echo-planar imaging sequences with 42 oblique slices (16° transverse to coronal) acquired in an interleaved order (1,500 ms repetition time (TR), 40 ms echo time, 1.5 mm isotropic voxels, 128 × 128 matrix, 192 mm field of view, 71° flip angle, acceleration factor 3, shift 2).
Area of acquisition	Functional slices produced only a partial volume for each participant, parallel to the hippocampus and covering the temporal and occipital lobes. Collecting a partial volume instead of the full brain allowed us to maximize spatial and temporal resolution over our a priori ROIs.

Diffusion MRI ☐ Used ☒ Not used

Preprocessing

Preprocessing software	Functional runs were corrected for slice-acquisition time and head motion, high-pass temporally filtered using a 128 s period cutoff, spatially smoothed using a 3 mm FWHM Gaussian kernel, and registered to each participant's high-resolution MPRAGE image using FLIRT boundary-based registration with B0-fieldmap correction.
Normalization	ROI analyses were performed in each participant's native space. For voxelwise analyses (in which we calculated the background connectivity of seed regions with all voxels in the partial volume), the reliability of the correlation maps for each seed ROI and condition was assessed after across participants after non-linear MNI registration using FNIRT.
Normalization template	For voxelwise analyses, data were normalized to the MNI152 template space, which had been resampled with interpolation to match the resolution of the functional data (1.5 mm isotropic).
Noise and artifact removal	Before measuring background connectivity, white matter and ventricle activity, along with six motion parameters, were regressed out of the preprocessed BOLD signal timecourses in a GLM for each run. Stimulus-evoked BOLD responses within each run were removed through a GLM containing FIR basis functions.
Volume censoring	Data acquisition in each functional run began with 6 s of rest to approach steady-state magnetization. These volumes (4 TRs per run) were removed prior to analysis. No other volumes were censored.

Statistical modeling & inference

Model type and settings	Stimulus-evoked activity was measured by a GLM containing FIR basis functions and local autocorrelation correction. Hippocampal-neocortical interactions were measured by ROI and voxelwise background connectivity. neural representations within the hippocampus and early visual cortex assessed based on multivariate pattern similarity. For each analysis, parameter estimates were averaged across runs for each participant with reliability tested across participants.
Effect(s) tested	Repeated-measures ANOVAs and paired-sample t-tests were used to compare background connectivity and pattern similarity for predictive and nonpredictive actions. Pearson correlations were used to compare the between-subject variability in RT, background connectivity, and pattern similarity. Two-sample t-tests were used to compare between groups of participants who were either consistent or inconsistent in their verbal predictions for nonpredictive actions.

Specify type of analysis: ☐ Whole brain ☐ ROI-based ☒ Both

Anatomical location(s)	Hippocampal subfields were anatomically defined on high-resolution T2-weighted images for each participant, using the automatic segmentation of hippocampal subfields (ASHS) machine learning toolbox and a database of manual medial temporal lobe segmentations. Early visual cortex for each participant was anatomically constrained to V1 and V2 (using anatomical masks generated with FreeSurfer), and functionally constrained to voxels reliably responsive to the experimental stimuli, as determined by an independent functional localizer.
Statistic type for inference (See Eklund et al. 2016)	Nonparametric randomization tests were performed for each voxel's connectivity using FSL Randomise.
Correction	All voxelwise statistics were corrected for multiple comparisons with threshold-free cluster enhancement (TFCE), resulting in a family-wise error rate of $p < .05$.

Models & analysis

n/a	Involved in the study
☐	☒ Functional and/or effective connectivity
☒	☐ Graph analysis
☐	☒ Multivariate modeling or predictive analysis

Functional and/or effective connectivity	Task-specific background connectivity was measured through a previously described approach (Al-Aidroos et al 2012 PNAS). After removing stimulus-evoked activity and confounding variables, background connectivity was measured as temporal Pearson correlations between concatenated background timeseries from each region and averaged across runs for each participant.
Multivariate modeling and predictive analysis	Multivoxel patterns for each cue-outcome transition were based on parameter estimates of BOLD response amplitude in an event-related GLM for each run. Multivariate pattern similarity in the hippocampus and early visual cortex was measured as Pearson correlations across voxels within each ROI.
